# Early Childhood Nutritional Status in CARICOM Countries: An Overview with respect to Five Nutrition Related Millennium Development Goals

**DOI:** 10.1155/2014/580928

**Published:** 2014-05-08

**Authors:** Pamela S. Gaskin, Anders L. Nielsen, Douladel Willie, Tara C. Durant

**Affiliations:** ^1^Faculty of Medical Sciences, University of the West Indies, Cave Hill BB11115, Barbados; ^2^Faculty of Medical Sciences, University of the West Indies, Mona, Jamaica; ^3^Faculty of Science and Technology, University of the West Indies, Cave Hill BB11115, Barbados

## Abstract

Previous reviews of nutritional status in children under 5 years describe the Caribbean grouped with Latin America. This paper focuses specifically on the Caribbean and the goals and targets of the Millennium Declaration that have bearing on childhood development. The results indicate that CARICOM countries have made progress in terms of child health as assessed by gross health indicators. Yet, the millennium generation experiences coexistence of undernutrition and overweight in early childhood. The associations of GNI with markers such as poverty indices are somewhat inconsistent with traditional findings and highlight a need to reassess the causes of infant mortality and low birth weight. However, a lack of systematic local data has hampered progress on an individual country basis. Interventions that deal more pointedly with country specific needs are required including those targeting obesity if the MDGs are to be attained by all member states.

## 1. Introduction

CARICOM countries like many other developing nations are in varying stages of an epidemiologic and nutrition transition [1], such that the traditional public health problems of infectious disease have decreased in prevalence, only to be largely replaced by the diseases of “life style” that are associated with Western-type diets and reduced activity [2]. A challenge facing the incoming millennium generation of the region is the easy availability of calories in the face of sometimes monotonous diets and reduced opportunity for exercise. The WHO proclaims that childhood obesity is one of the most serious public health challenges of the 21st century, with close to 35 million overweight children under the age of five in developing countries [3].

Undernutrition in early childhood is associated with developmental deficits of reduced cognitive [5] and psychosocial [6] functioning as well as later physical and work capacity [5]. In addition, many children who experience undernutrition will be at increased risk of developing chronic diseases [7]. Nutritional status in children clearly has implications for the future of the individual as well as for these nation states as a whole. Tracking these patterns over time is important if policy tailored to the unique needs of the different countries is to be devised. The Millennium Declaration [8] signed by 189 countries, including CARICOM in September 2000, set out to create an environment which is conducive to development and the elimination of poverty. The goals and targets are interrelated and several have bearing on childhood nutritional status.

The first five millennium development goals (MDG) [8] have bearing on children's nutritional status, health, and development. These goals are to eradicate extreme poverty and hunger, achieve universal primary education, promote gender equality and empower women, reduce child mortality, and improve maternal health.

The MDGs included these gross health indicators for monitoring progress: infant mortality, the under-five mortality rate, and antenatal care coverage. Associated recommendations were to prevent low birth weight, improve early feeding practices, begin breastfeeding within one hour of birth, exclusive breastfeeding for the first six months of life, and timely and appropriate complementary feeding from six months of age-continued breastfeeding up to two years of age.

In this paper, we describe the nutritional status of children under 5 years in CARICOM countries, reported since 2000, and seek to assess the current situation in relation to the first five MDGs, which contain aspects that pertain to children's nutritional status. In addition, we reviewed available work on childhood obesity in the Caribbean because of the WHO proclamation that childhood obesity is one of the most serious public health challenges of the 21st century, with close to 35 million overweight children under the age of five in developing countries. Reviews of this nature have been conducted that group the Caribbean together with Latin America. However, this paper focuses specifically on the Caribbean and should assist with planning and policy making that caters to the unique characteristics of these small states that form CARICOM.

## 2. Materials and Methods

### 2.1. Literature Search

A PubMed and Google Scholar search up to 2010 was made using the following keywords: “food,” “childhood,” “CARICOM,” “Caribbean,” “under-nutrition,” “overweight,” “obesity,” “low birth weight,” “infant mortality,” “breast feeding,” “maternal education,” “mothers,” “maternal,” “education,” “child,” “stunting,” “height for age,” “weight change,” “developing countries,” “West Indian,” “Millennium Development Goals,” “weight for height,” and “nutrition.” The reference lists of articles retrieved from PubMed and Google Scholar were also reviewed.

### 2.2. Study Selection

Publicly available literature for original data on child development in CARICOM countries published since the year 2000 was used. We included observational and experimental studies. Data from published reports from WHO, UNICEF, CIA World Fact Book, Caribbean Food and Nutrition Institute, and UNESCO were used.

### 2.3. Methodology

Information on child growth and wellness indices was used as a basis on which to track early childhood development. Feeding practices were used to estimate the progress of interventions aimed at eliminating poor nutritional status [5, 9]. We specifically collated information on infant mortality, low birth weight, stunting, wasting, overweight, and infant feeding. Where original data was not available we used estimates collated by international health and information agencies [4, 10]. From the literature review, we found primary data from surveys for Jamaica, [11] Trinidad and Tobago [12], and Guyana [13] in the form of multiple indicator cluster surveys (MICS). Most of the other data used in calculations was taken from UNICEF, [4] other international agencies, or from country reports that tabulate health and economic data [10, 14]. The three countries that had published surveys, Jamaica, Trinidad and Tobago, and Guyana, were used to represent more detailed descriptive comparisons for each indicator. We added Barbados and Haiti to represent the range of gross national income** (**GNI) across the region. The GNI for Barbados was estimated from gross domestic product (GDP), 2007 [15]. As an initial step, we examined the relationship of GNI and infant mortality in the CARICOM states to determine whether the data for the region presented by UNICEF followed expected trends [16]. In addition to using individual country data, a summary statistic for developed countries was included. Montserrat was omitted from the analyses because of the disruption by the volcano.

As mentioned before, the MDGs that have bearing on child nutritional status, health, and development are as follows:

 Goal 1: eradicate extreme poverty and hunger,
 Goal 2: achieve universal primary education,
 Goal 3: promote gender equality and empower women,
 Goal 4: reduce child mortality,
 Goal 5: improve maternal health.

For each goal, indicators for monitoring progress were identified for analysis in the results [17]:

 Goal 1: eradicate extreme poverty and hunger
   Indicator 1.8: prevalence of underweight children under five years of age
 Goal 2: achieve universal primary education
   Indicator 2.1: net enrolment ratio in primary education
 Goal 3: promote gender equality and empower women
   Indicator 3.1: ratios of girls to boys in primary, secondary, and tertiary education
 Goal 4: reduce child mortality
   Indicator 4.1: under-five mortality rate, 4.2: infant mortality rate
 Goal 5: improve maternal health
   Indicator 5.5: antenatal care coverage.

Additional gross health indicators were employed to examine nutritional status in the separate states of CARICOM that were not specifically identified by the MDG targets or indicators. These are interrelated; for example, neither low birth weight nor infant feeding practices are specifically named as an MDG, but both relate to infant mortality and antenatal care which are clearly identified MDG indicators for monitoring progress. Therefore, these common health indicators were noted under the specific MDG and indicators that they correlate with in the results.

## 3. Results

The results are presented with respect to overweight and obesity in the Caribbean region and in accordance with the aforementioned MDG targets and recommendations. For the purposes of this paper, overweight in children is defined as a* Z*-score value > 1 s.d. above the WHO/NCHS mean weight-for-height while obesity in children is defined as weight-for-height* Z*-scores > 2 s.d. [18].

### 3.1. Overweight

Using data compiled from various sources including some unpublished country surveys, the Caribbean Food and Nutrition Institute (CFNI) compiled and published in 2001 regional estimates of overweight among the children ages of 0–5 years. They report an overall prevalence of 3–6% and from clinic data a trend of increasing prevalence over a ten-year interval [19]. Information on Trinidad and Haiti was available from other sources which used WHO definitions of overweight and obesity.

#### 3.1.1. Overweight in Selected Caribbean Regions

The estimate for Jamaica for overweight prevalence among children of 0–5 years from a 1993 survey was 6% [20]. A lower estimate of 4% was arrived from an unpublished survey conducted in 1998 by CFNI [19].

Data from a 1987 national nutrition survey recorded the prevalence of overweight of approximately 8.9% and obesity at 1.9% among children 12–36 months old [16]. However, there were significant rural urban and SES differences [21]. Another author using slightly older children from this same sample (12–59 months old) estimated overweight prevalence at 3%. However, the definition of overweight used here was what was used as obesity by the previous author [20]. The overweight estimates quoted in the CFNI 2001 report (3%) in the 0–5-year age group are in keeping with the latter study but a source is not identified [19].

For Guyana, there was an estimated overweight prevalence children 1–5 years old of 2.3% from a 1981 survey [20]. The CFNI estimate from an unpublished survey conducted in 1981 of 1% in the 0–4-year age group is considerably lower [19].

The prevalence of overweight in Haiti for 1994-1995 defined in a national survey as >1 SD above mean weight-for-height in children 12–59 months of age was approximately 5.7% and obesity was 1.4% [16]. These data show no difference in prevalence by geographical setting, SES, sex, nor level of maternal education [21]. Among a slightly younger group from the same sample (3–59 months) another author estimated the prevalence of overweight as 2.8% defined as >2 SD above mean weight-for-height. This represents an increase of 2 percentage points since 1987 [20].

The incidence of overweight among children of 0–5 years of age at 1981 was an estimated 3.8% for Barbados in a national nutrition survey. Obesity was defined as >120% of the weight for length using the Gomez classification. All other estimates for Barbados quote this source [22].

### 3.2. MDG Goal 1: Eradicate Extreme Poverty and Hunger

 Indicator 1.8: prevalence of underweight children under five years of age.

#### 3.2.1. Stunting

The prevalence and demographic distribution in the Caribbean of stunting is not well described. Notably, levels in the Caribbean of wasting and stunting are below those for other developing countries. However, there appears to be no trend with GNI. Half of the CARICOM countries report no undernutrition among children under five years at all. The countries that report wasting also report stunting but stunting is most prevalent (Table 1) and is high in Haiti where levels approach the mean for developing countries [4]. Levels in Belize and Guyana are also of concern.

### 3.3. MDG Goals 2 and 3: Achieve Universal Primary Education and Promote Gender Equality

 Indicator 2.1: net enrolment ratio in primary education
 Indicator 3.1: ratios of girls to boys in primary, secondary, and tertiary education.

Net enrollment in primary school was high in Guyana in 2006 with no significant gender bias (boys 96.3%; girls 96.0%). There was a relatively sharp decline for entrance to secondary school (boys 66.1%; girls 72.6%) [13].

For Jamaica, net enrollment in 2005 was 89% with no gender bias with high rates of transition to secondary school (97% both boys and girls) [11].

For Trinidad, 2006, the net primary school enrollment rate was 82% with equal numbers of boys and girls. Transition to secondary school was 97.9% [12].

For the period 2003–2008, UNICEF reports Barbados net primary enrollment at 97% and transition to secondary school (boys 88%; girls 93%). For Haiti, primary school enrollment was at 50%. No estimates were available for transition to secondary school but attendance rates were low (boys 18%; girls 21%) [4].

#### 3.3.1. Primary Education and Infant Mortality

Maternal education appears to be of negligible importance for the region because it is only associated with mortality when the mother has not finished primary school. This was observed only in Haiti and Guyana [11, 13].

### 3.4. MDG Goal 4: Reduce Child Mortality

 Indicator 4.1: under-five mortality rate, 4.2: infant mortality rate.

**Figure 1:**
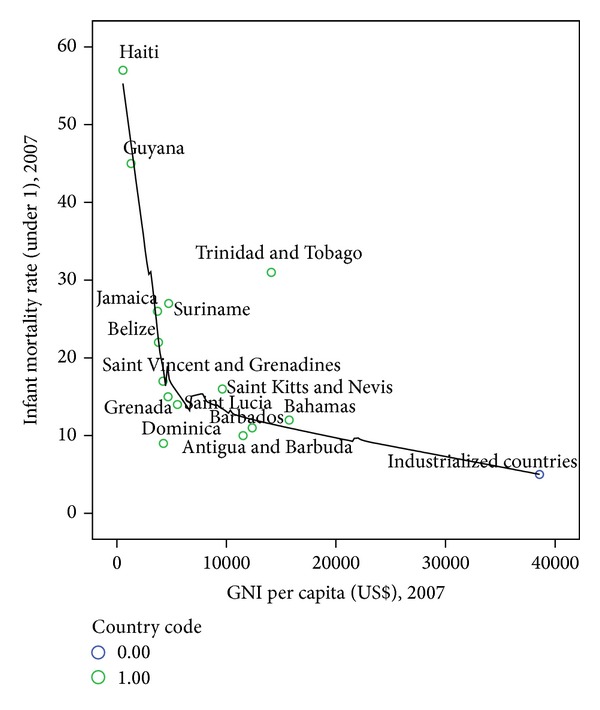
Incidence of infant mortality (IM) in Caribbean countries in relation to their gross national income (GNI) [23].

There was an inverse relationship of infant mortality to GNI (*P* = 0.051) for CARICOM countries (Figure 1). The curve flattens as GNI exceeds 5000 $ USD per capita [23].

#### 3.4.1. Countries with Multiple Indicator Cluster Survey (MICS) and Infant Mortality

In Jamaica, infant mortality was estimated at 26 per thousand. Infant and under-5 mortality rates were lowest in the rural areas and highest in other towns (excluding Kingston and the metropolitan area) [11]. The infant mortality rate for Trinidad and Tobago was 29 infant deaths per thousand. The under-five mortality rate is estimated to be around 35 per one thousand live births. There are regional differences between Tobago at 48/1000 and the South West region at 16/1000 [12].

In Guyana, the infant mortality rate in 2003 stood at an estimated 37 per thousand. Infant mortality was 12 percent higher among rural compared to urban children. Child mortality was lower among east Indian children than among children of the other ethnicities [13].

Infant mortality rate in Barbados is estimated at 12 deaths per thousand live births for 2009 [10]. The same figure was quoted at 2005 [14].

Infant mortality rate for Haiti was estimated for 2009 at 60 deaths per thousand live births. This is a reduction from an estimated 76 in 2003. Both estimates are prior to the earthquake of 2010 [10].

#### 3.4.2. Low Birth Weight

**Figure 2:**
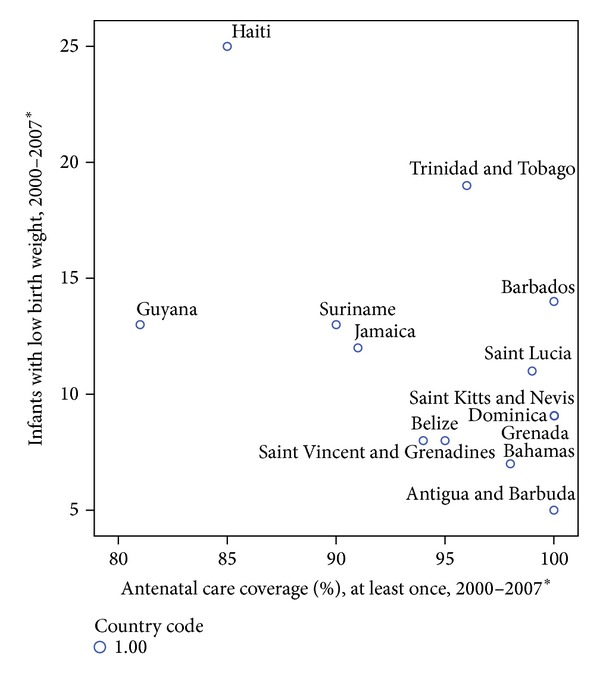
Percentage of infants with low birth weight in CARICOM countries by antenatal care coverage [23].

A child is almost ten times more likely to die if they are severely underweight than if they are of average weight for their age [24]. This links low birth weight with infant mortality. There is a general increase in LBW with increasing GNI with Haiti having highest incidence of LBW, likely due to intrauterine growth retardation (IUGR) [25]. The picture is similar though not as consistent as that seen with infant mortality. For example, the prevalence in Barbados does not fit the general Caribbean pattern so that despite high GNI and antenatal care the incidence of low birth weight is higher than for several lower income Caribbean countries (Table 2; Figure 2).

#### 3.4.3. Low Birth Weight in Selected Caribbean Regions

Results from the Jamaica MICS estimated low birth weight (birth weigh less than 2500 grams) at 12 percent of live births. This is a reliable estimate since ninety-seven percent of infants are weighed at birth [11].

Trinidad and Tobago reported LBW from the MICS at 18.8% as 89.8 percent of live births were weighed at birth. There was variation by socioeconomic status. The highest percentages of LBW infants occurred among the poorest (20.6) and upper middle quintile (21.5) groups while the other quintiles represented the lowest percentages of LBW (16.4, 18.2, and 17.1, resp.) [12].

In Guyana, low birth weight was estimated at 19 percent. There was geographic variation with higher incidence in the interior among Amerindian women (24 percent) [13]. Interestingly, Guyana recorded a 7 percent increase in low birth weight between 2000 and 2006. Seventy-eight percent of births in Guyana were weighed at birth. Mothers' level of education and household wealth did not appear to have much impact on low birth weight status among infants.

Haiti occupies the top of the rank with respect to prevalence of LBW in the Caribbean; this is in keeping with its low GDP and uneven distribution of wealth. Mothers likely have restricted access to food in several instances resulting in IUGR. Surveys documenting demographics and distribution are lacking [13].

No recent detailed studies of low birth weight were identified for Barbados. There is some speculation that higher rates in Barbados reflect teenage pregnancies; this seems unlikely given that rates of teenage pregnancy are low in Barbados. An alternate explanation of higher rates of pregnancy among older women leading to greater IUGR and LBW seems more credible, but the supporting data has not been collected. According to Nation Master.com [26], 95% and 24% of children are delivered by a skilled attendant in Barbados and Haiti, respectively, so this would reflect, though not exactly, the number of weighed births in these countries. This suggests that the estimates of low birth weight at least for Barbados are likely to be close to the true value.

#### 3.4.4. Early Feeding Practices

Underweight in children can be prevented through initiatives promoting breastfeeding, complementary feeding, micronutrient supplements, and social protection mechanisms [24].

*Breastfeeding*. Only five of the CARICOM countries have not formally reported on breastfeeding in the recent past. In Haiti, just under half of mothers meet the recommendation to exclusively breastfeed for six months (Table 3). In those countries that report breastfeeding with supplementation at 6–9 months, the percentage of children breastfeeding with supplementation is higher than of exclusive feeding (Table 4). This represents a trend of an earlier introduction of complementary foods than recommended. Haiti has the highest percentage of mothers (35 percent) who meet the recommendation for breastfeeding up to 24 months (Table 4).

### 3.5. MDG Goal 5: Improve Maternal Health

Indicator 5.5: antenatal care coverage.

*Antenatal Care and Low Birth Weight. *When GNI is controlled for antenatal care, the expected inverse relationship of LBW to GNI is revealed (*P* = 0.064).

## 4. Discussion

Nutritional status is the best global indicator of well-being in children and is an indicator of the overall well-being of a society [27]. Children are growing and are therefore more susceptible to environmental change, so growth is a good indicator of nutritional status among children. For this reason, child growth and wellness indices are frequently monitored and form a useful basis on which to track development [5, 9].

There was a trend of decreasing infant mortality with increasing GNI across the region. Trinidad and Tobago was a notable exception [12]. The detailed country descriptions suggest that the urban, rural, and geographic regional differences that presumably correspond to socioeconomic status (SES) were also loosely associated with income, but the patterns are not consistent from country to country with rural urban patterns moving in opposite directions in some cases.

In the five countries selected for more detailed comparisons, infant mortality rate was calculated in MICS using the Brass method [28]. This led to a trend of the higher estimates of child mortality to previous estimates for these countries. This indicates that the UNICEF estimates may all be low in other countries. It should be noted that data on specific causes of death has not been presented mostly because indirect means are used to estimate infant mortality. Evidence on ethnic differences in the epidemiology of infant mortality in the United States indicates that differences in social circumstances are likely to be associated with a different distribution of early mortality [29]. This may partially explain the high and regional variation of rates experienced in Trinidad and Tobago.

Child mortality rates in both infants and those under five are critical indicators of well-being of children. In the Caribbean, rates are very moderate to low. Haiti has the highest rates in the region and ranks the 48th in the world, whereas Barbados ranks at 140, with rates much closer to rates of 8 and 6 per thousand live births seen in the US and UK, respectively. Rates in Trinidad and Tobago are surprisingly high, given the standard of living and gross national income (Figure 1).

Information on the causes of infant mortality is often not readily available in the Caribbean. So, although there is an obvious macroscopic association of poverty with child mortality (Figure 1), the patterns are not so obvious at the county level. When GNI increases to around $5000 USD per capita, its correlation with infant mortality is no longer evident. This suggests that decreasing poverty as an intervention to reduce infant mortality has limited effect after this point at the country level.

Since the infant mortality rates in many of the Caribbean countries approach those of the UK and USA, we can assume that the broad interventions applied in the mid-twentieth century have worked to produce as much improvement in infant mortality as they are likely to. Clearly, different types of study must be conducted in the region if we are to understand and address the causes of infant mortality especially in the more developed of the territories where the broad indicators like poverty and maternal education have lost some of their sensitivity. The high rates of infant mortality in Guyana and Haiti remain a concern.

### 4.1. Low Birth Weight

The prevalence patterns of low birth weight for most of the countries have remained virtually static since the mid-nineteen-eighties (Walker 1989). This may be attributed to a lack of reliable estimates in some cases. In Guyana and Trinidad and Tobago, ethnic groups may have an effect in that around half of the Trinidad population and one-third of the Guyana population are of Indian extract, known to have smaller babies [30]. Nevertheless, the regional differences appear to represent to some extent women with different access to education and antenatal care. Although there are some aberrations such as St. Lucia, the obvious inverse associations of reduced antenatal care and of education when less than primary level is attained indicate that addressing vulnerable women remains an important strategy for regional development.

In the developing world, estimates of incidence of low birth weight are often hampered by infants not being weighed at birth. Sometimes prevalence is estimated from biased nonrepresentative samples taken from health facilities or may be estimated from maternal recall. However, because there are skilled attendants at most births in CARICOM countries, this is less of a problem than in other developing countries.

As a consequence of the lack of studies that elicit details on reasons for low birth weight, it is possible only to speculate about the reasons for the variations observed. One might expect low birth weight to consistently decrease in the face of improved antenatal care; however, Figure 2 demonstrates that this indicator like infant mortality paradoxically can rise when conditions in a country become quite good such that better antenatal care prolongs pregnancies that formally would not have been viable. A higher proportion of these infants succumb to the problems of being born early for gestational age, thereby increasing both infant mortality and incidence of low birth weight [31]. This effect is likely more pronounced in states like Barbados where there are fewer resources to sustain premature infants than is ideal compared to the developed world.

### 4.2. Stunting

Linear growth retardation is known to affect about 30% of children in the developing world and to have long term effects on development [32, 33]. Early childhood stunting is associated with poor school achievement and reasoning in young adults [33, 34]. Children stunted before age 2 years have poorer emotional and behavioral outcomes in late adolescence than nonstunted children [35]. However, studies have demonstrated that some of the effects can be ameliorated with early intervention [35].

Although, by comparison to other countries in the developing world, the rates of stunting in the Caribbean are relatively low, in absolute terms, this represents quite a large burden on these vulnerable states. Of the approximately 16.7 million children under five years in CARICOM countries, an estimated 142,750 are wasted and an alarming 402,008 stunted [4].

This continued high prevalence of stunting in some territories is of great concern. It highlights the fact that interventions such as those conducted in Jamaica in the late twentieth century still have a high degree of relevance for many pockets of children in the region. Overall prevalence in the Caribbean and gross numbers of stunted children are low compared to regions like Eastern Africa and Asia, where as recently as 2005 prevalence ranged from 43.7% to 34.9%, respectively, of the 5 years and under population.

Trinidad and Tobago remains an anomaly with respect to under-five growth and development. They record significant negative measures of every childhood growth and development indicator despite having concurrently one of the highest GNIs in the region (Tables 1-2). Other countries in the eastern Caribbean have done better with apparently fewer resources. The lack of a direct relationship between GNI and measures of childhood undernutrition is possibly explained by differences in public health management of the problem and due to historical differences in economic and natural disaster misfortunes, in places like Haiti. These challenges need to be addressed, for the children currently living in these circumstances.

### 4.3. Overweight

While for most of the developing world obesity does not appear to be a public health problem among preschool children, in a number of countries in Latin America and the Caribbean, levels equal those of the USA [16]. The WHO proclaims that childhood obesity is one of the most serious public health challenges of the 21st century, with close to 35 million overweight children under the age of five in developing countries. Of concern in this region as elsewhere is the coexistence of undernutrition and high levels of overweight [36]. Grappling with this paradox is a troubling prospect for a region with scarce resources. It is nevertheless difficult to find credible data for CARICOM countries that confirms this especially among children under 5 years. This clearly points to a large gap in the research. The data on overweight and obesity prevalence and incidence among Caribbean children are even sparser than those on undernutrition. The few measures that are available are not on exactly comparable groups and different definitions of overweight are used [16, 20]. This makes the picture quite unclear. The necessary measurements have not been routinely collected for most countries up to 2012 and few population surveys have been conducted [19]. Nevertheless, there is some evidence, for example, from Haiti that prevalence of overweight is increasing and anecdotal evidence from the other territories that this is a growing problem. This is important because early growth is associated with the prevalence of obesity later in the life course [37].

### 4.4. Breastfeeding

Breastfeeding is known to be associated with many positive childhood outcomes including protection against overweight [38, 39], although there is debate as to the reasons for the positive associations [40]. Early introduction of solid foods on the other hand appears to potentiate negative outcomes including allergy [41] and obesity. Mothers and grandmothers are sometimes known to hold beliefs that may promote development of overweight [42]. This is important in many CARICOM countries where the prevalence of obesity and associated cardiovascular disease has risen drastically in the last 20 years [2, 43]; importantly, rates among children are also rising [44, 45].

Exclusive breastfeeding before six months is recommended as it is thought to have a protective role in conditions spanning asthma to overweight [46]. WHO recommendations for adequate feeding seek to have infants and toddlers exposed to optimal nutrition as a means of reducing the burden of disease among coming generations. How well the Caribbean has heeded these recommendations is an indicator of likely progress with helping children to exploit their developmental potential. Cultural norms, social pressure, and working mothers are some of the factors that impinge upon this important nutrition intervention.

There is scant information on early childhood feeding practices in the region; as a whole, many of the higher income countries like Barbados and Bahamas do not even report basic statistics. Haiti reports that 87% of mothers breastfeed at some point; 35% of them are still breastfeeding when the child is 20–23 months, in keeping with recommendations. However, only 56% of mothers exclusively breastfeed up to six months. So even where resources are scarce there is a trend of early introduction of complementary food. This propensity for introduction of early solid foods is of concern as it appears to be most prevalent in those countries like Barbados where adult obesity and chronic cardiovascular disease are major problems, (unpublished data; Beverly Stanford, National Nutrition Centre, Barbados). It is not currently known whether heavier breastfed babies are at higher risk of becoming overweight than slimmer breastfed babies; however, it is known that breastfed babies have a lower risk of later becoming overweight than formula-fed babies [47].

### 4.5. Interventions

Most of the substantive interventions carried out in the Caribbean region were conducted in Jamaica. This section will briefly examine some of those studies [6].

One major intervention study in Jamaica was initiated with a cohort of stunted children 9–24 months old from inner city Kingston. Children received nutritional supplementation, stimulation (mothers playing with homemade toys), or both. Initially, both interventions showed positive effects on development [48] but by age 17 to 18 years only the effect of stimulation remained [49]. Stimulation administered to stunted children in early childhood was associated with reduced cognitive and educational deficits compared to controls in late adolescence [6]. Children stunted in early childhood had significant more anxiety than their nonstunted counterparts but those who received early stimulation were somewhat better than controls but remained at a disadvantage with respect to emotional and behavioural outcomes in late adolescence [35].

This important work out of Jamaica demonstrates that the effects of stunting can be alleviated with feasible interventions using materials available to poor working class parents.

We were unable to identify a regional study that successfully intervened in early childhood overweight or obesity. Some school based interventions in children and adolescents have shown promise whereby children have been shown to change habits associated with development of overweight [45, 50]. However, in general in the world most school based interventions to reduce BMI at the population level have been unsuccessful [50].

### 4.6. Education and Gender Equality

The states of CARICOM except for Haiti have in most instances achieved the MDGs of primary education goals and gender equality. Although enrolment in primary schooling is high, transition to secondary school is less consistent. In addition because education is a major tool of empowerment the trend of equal opportunity for schooling both at the primary and secondary levels demonstrates commitment to the goal of promoting gender equality.

## 5. Conclusion

The nutritional status of Caribbean children under 5 years is relatively good compared to children in other developing nations. CARICOM countries by and large have reached the millennium development goals with respect to early childhood nutritional status, although there are lingering pockets of undernutrition in some Caribbean states. There is also emerging obesity in this age group. The country level inconsistencies may be explained by geography, ethnicity, and schooling. Some of these differences may be due to the transition from low income to high income country lifestyles. It appears that the large scale poverty alleviation interventions such as lowering of childhood mortality and low birth weight have reached their peak of efficiency in the region. Consequently, to improve the health status of children under 5 years, it is important to explore the drivers for disparities on an individual country basis. Research and interventions that deal more pointedly with country specific needs are required including those targeting obesity if the MDGs for CARICOM are to be attained by all member states.

## Figures and Tables

**Table 1:** Early childhood health indicators ranked by gross national income.

Country or region	GNI per capita (US$), 2007	Percentage of infants with wasting, 2003–2008 (NCHS/WHO)	Percentage of stunted infants, 2003–2008 (NCHS/WHO)
Haiti	560	10	29
Guyana	1300	8	17
Jamaica	3710	2	4
Belize	3800	2	22
Suriname	4730	5	11
Trinidad and Tobago	14100	4	4
Developing countries	2405	11	30

[4].

**Table 2:** Low birth weight among infants in CARICOM countries ranked by gross national income.

Country or region	GNI per capita (US$), 2007	Percentage of infants with low birth weight, 2000–2007
Haiti	560	25
Guyana	1300	13
Jamaica	3710	12
Belize	3800	8
Saint Vincent and the Grenadines	4210	8
Dominica	4250	9
Grenada	4670	9
Suriname	4730	13
Saint Lucia	5530	11
Saint Kitts and Nevis	9630	9
Antigua and Barbuda	11520	5
Barbados	12356	14
Trinidad and Tobago	14100	19
Bahamas	15730	7

[4].

**Table 3:** Exclusive breastfeeding up to six months in CARICOM countries.

Country or region	% of children (<6 months ) (2000–2007) who are exclusively breastfed
Bahamas	—
Barbados	—
Belize	10
Dominica	—
Haiti	41
Jamaica	15
Saint Kitts and Nevis	56
Saint Lucia	—
Saint Vincent and the Grenadines	—
Trinidad and Tobago	13

[4].

**Table 4:** Breastfeeding with supplemental feeding in CARICOM countries.

Country or region	% of children (6–9 months) (2000–2007) who are breastfed with complementary food	% of children (20–23 months) (2000–2007) who are still breastfeeding
Bahamas	—	—
Barbados	—	—
Belize	—	27
Dominica	—	—
Haiti	87	35
Jamaica	36	24
Saint Kitts and Nevis	—	—
Saint Lucia	—	—
Saint Vincent and the Grenadines	—	—
Trinidad and Tobago	43	22

[4].
